# Supplementary material for: Mitochondrial genome copy number measured by DNA sequencing in human blood is strongly associated with metabolic traits via cell-type composition differences
Source: Hum Genomics. 2021 Jun 7;15:34. doi: 10.1186/s40246-021-00335-2 (PMC8185936; doi:10.1186/s40246-021-00335-2)
Supplement: Supplementary file 1 — Additional file 1:. PDF format. Supplementary Figures and Small Supplementary Tables. Contains Figures S1-S8 and Tables S2-S4, S9, and S11. [file 40246_2021_335_MOESM1_ESM.pdf]

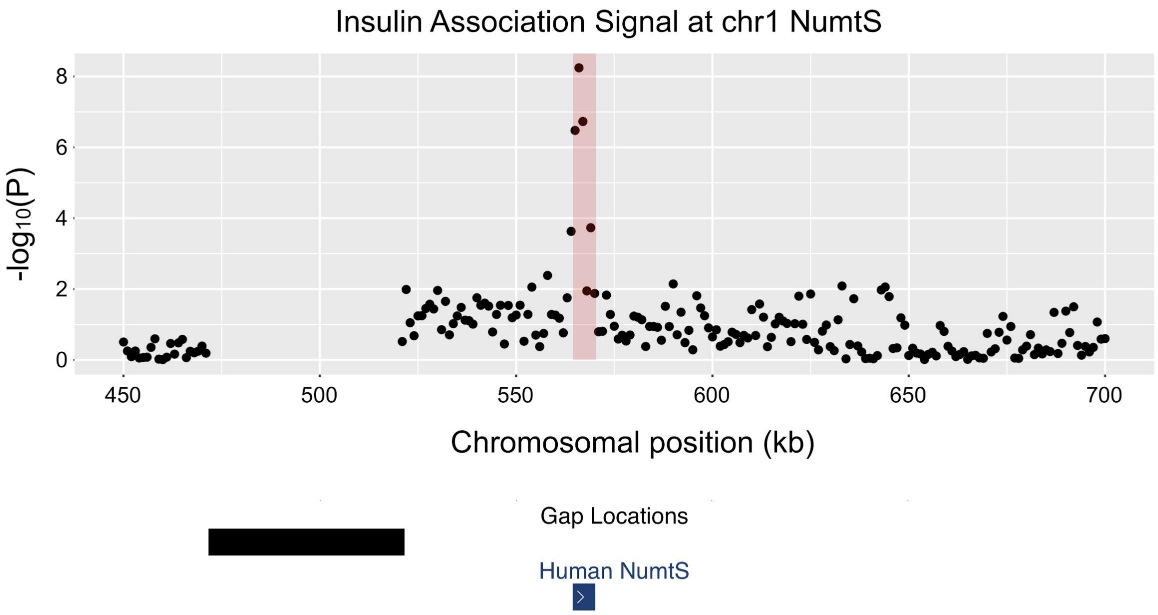

Figure S1. Insulin association signal at chromosome 1 NumtS. Manhattan plot from an analysis of copy number variation at nuclear mitochondrial insertion sites (NumtS) using CNVnator read-depth measurements in 1 kb genomic windows, using an initial set of 2,049 samples from an earlier data freeze analyzed for an unrelated CNV association study (manuscript under review - preprint doi: 10.1101/2020.12.13.422502). Shown at the bottom are two tracks from the UCSC Genome Browser. The black track represents an assembly gap upstream of the association signal, and the blue track shows the location of the NumtS region where the association peak is located.

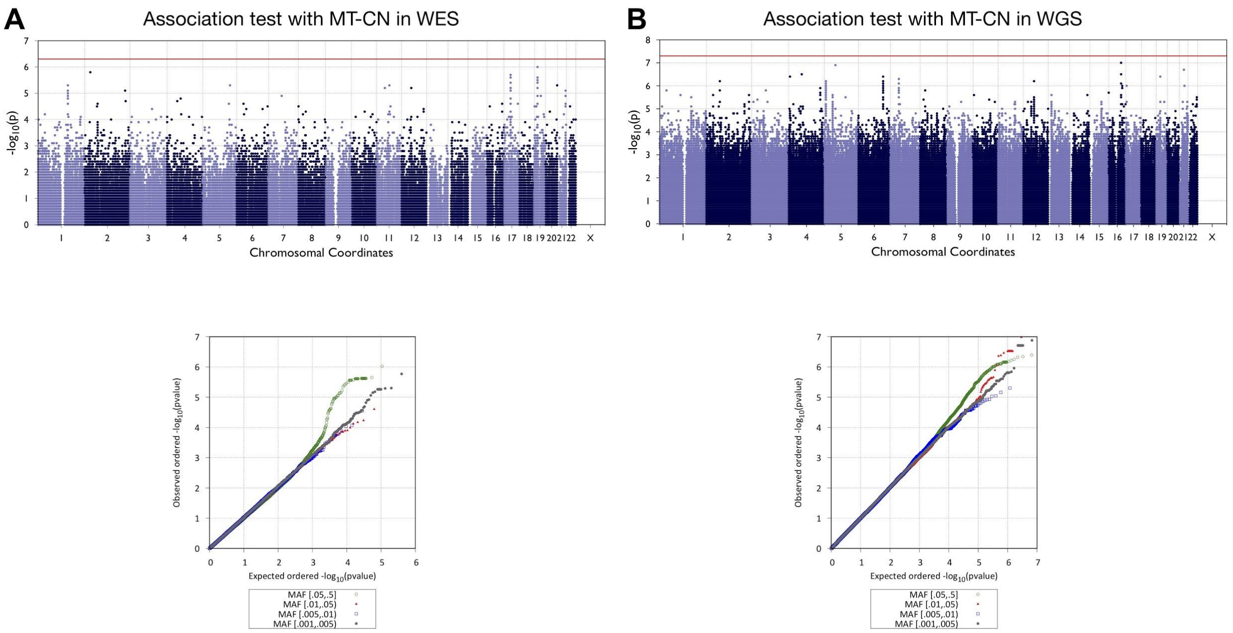

Figure S2. Joint (METSIM and FINRISK) single-marker association tests with MT-CN in WGS and WES data. (A) Manhattan plot and quantile-quantile (QQ) plot for an exome-wide association test of normalized, WES-measured MT-CN using WES genotype data (N = 19,034). The red line represents an exome-wide significance level of 5×10^{-7}; no tested markers achieved this level of significance. The QQ plot is separated by minor allele frequency bin, as indicated by the colors and shapes of the points. (B) Manhattan plot and quantile-quantile (QQ) plot for a genome-wide association test of normalized, WGS-measured MT-CN using WGS genotype data (N = 4,149). The red line represents a genome-wide significance level of 5×10^{-8}; no tested markers achieved this level of significance. The QQ plot is separated by minor allele frequency bin, as indicated by the colors and shapes of the points.

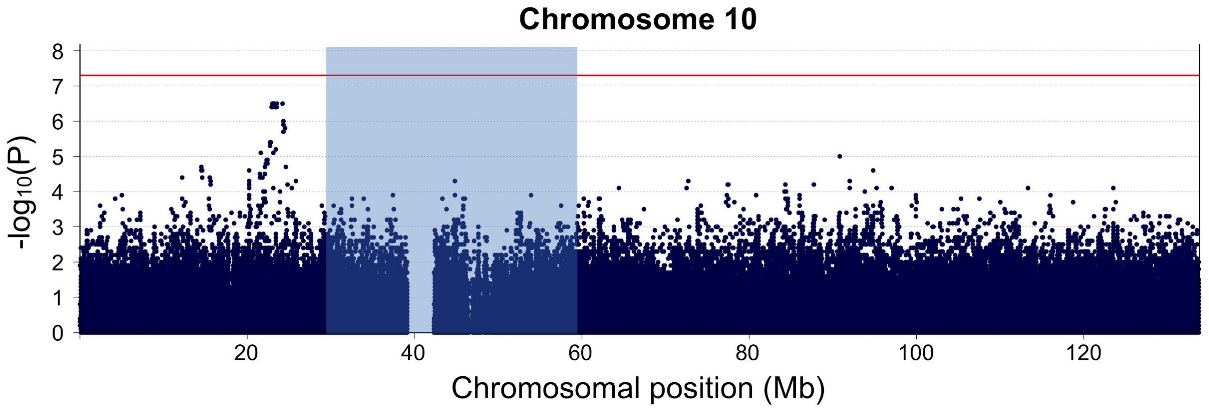

Figure S3. Single-marker association test results at chromosome 10 QTL identified by Curran *et al.* Chromosome 10 Manhattan plot for association with WES-estimated MT-CN in Finnish imputed array data from METSIM (N = 9,791). The blue highlighted region represents the approximate location of the linkage peak reported for MT-CN in Curran *et al*., 2007. This region is larger than the reported region in the Curran study (~30 Mb vs. ~24 Mb) because the exact coordinates of the 1-LOD support interval were not reported explicitly, so the genetic coordinates were approximated from a figure and converted to physical coordinates using a genetic map from HapMap Phase 2. Some error may have been introduced due to differing populations between the HapMap data and the Mexican-American samples used by Curran.

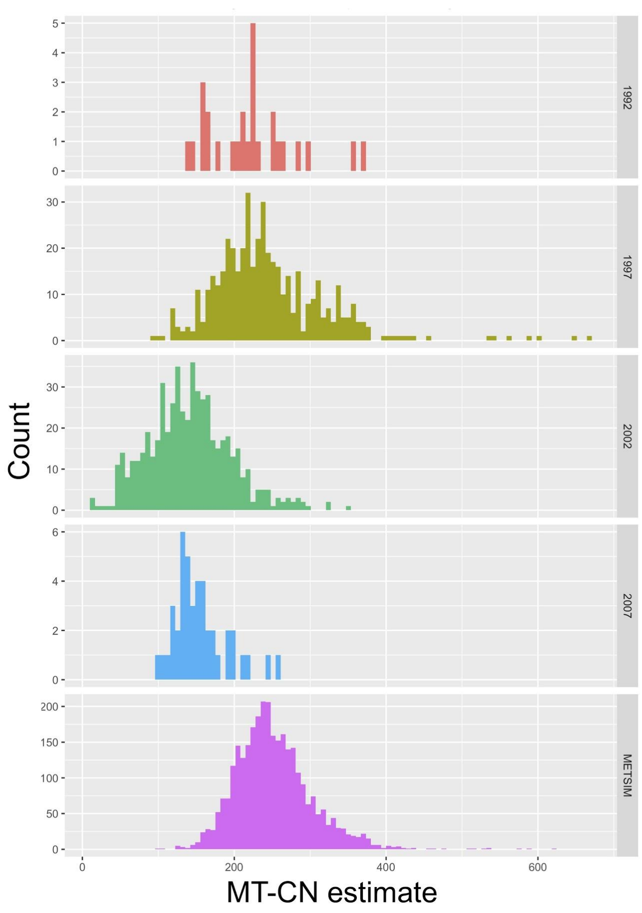

Figure S4. Raw WGS-based MT-CN estimate distributions for METSIM and all four FINRISK surveys. Facet labels “1992”, “1997”, “2002”, and “2007” refer to individual FINRISK survey years.

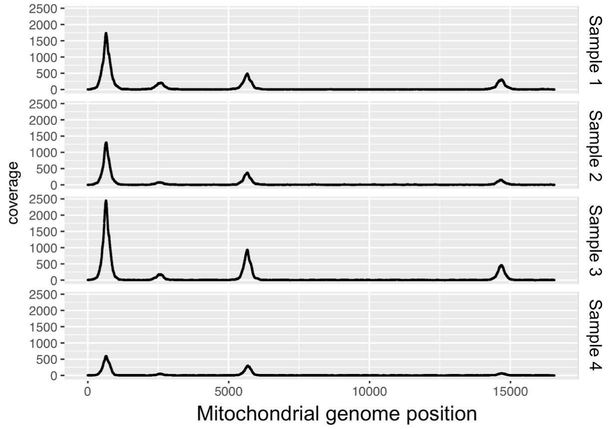

Figure S5. Nonuniform WES coverage across the mitochondrial genome for four representative samples.

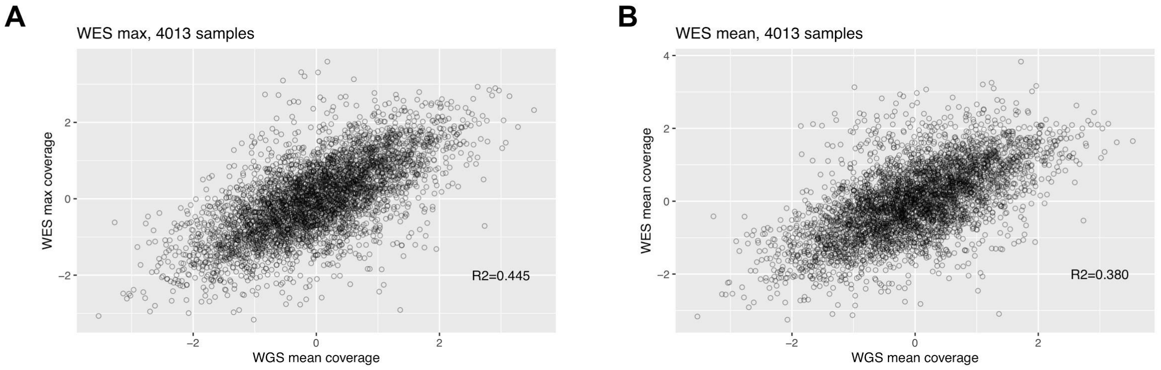

Figure S6. Comparison of two methods of summarizing nonuniform mitochondrial coverage in WES into a single measurement. Both panels show the correlation with WGS mean coverage for the 4,013 samples for which both WGS and WES data were available. (A) shows the results of summarizing WES mitochondrial coverage using the maximum coverage value across the mitochondrial chromosome while (B) shows the results of using the mean mitochondrial coverage instead. R^2 values are shown on each panel.

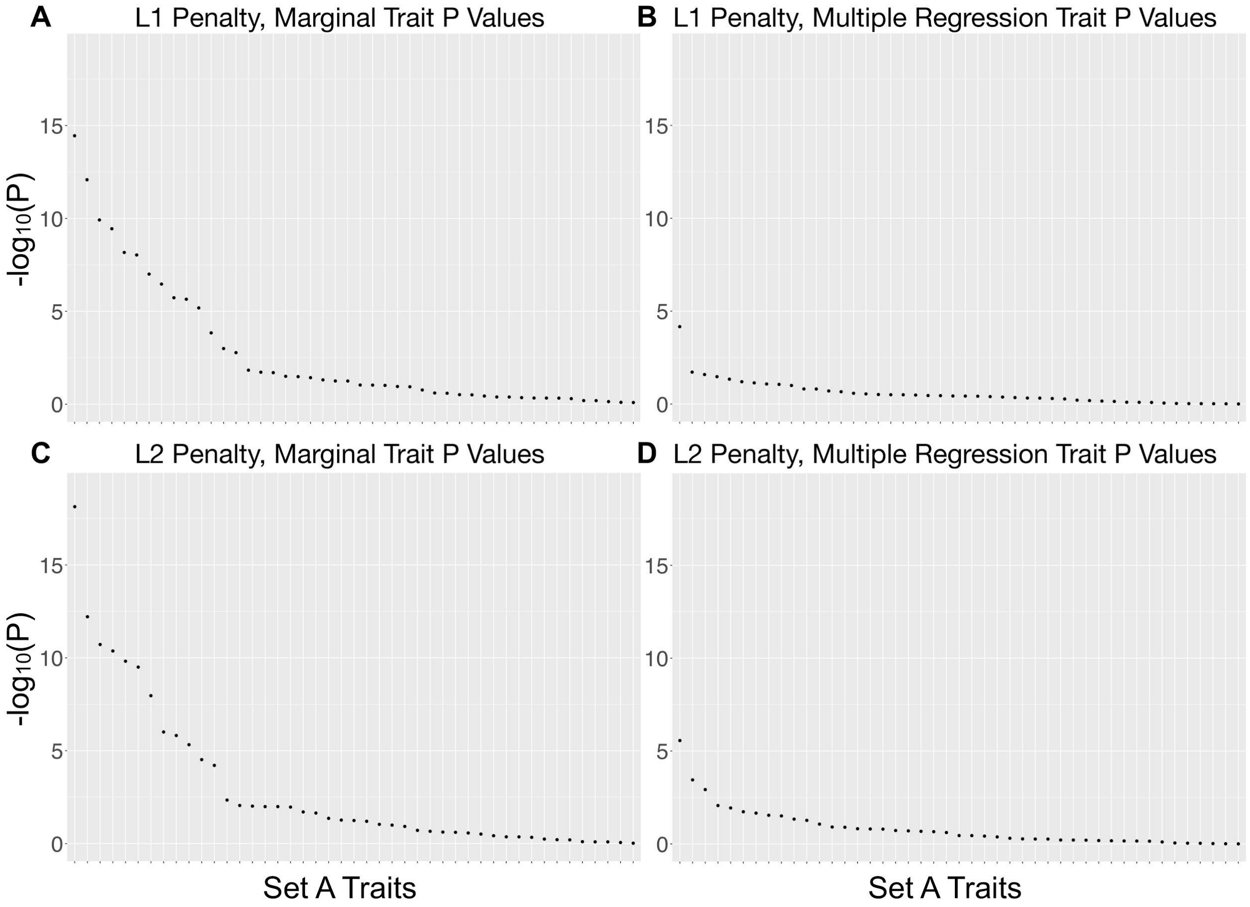

Figure S7. Associations between Z and traits in set A in imputed data. P values computed from linear regression using the output from a randomly chosen run of multiple imputation of phenotype data. (A) and (C) show the marginal P values of the traits in set A from regressions of Z onto each trait separately, while (B) and (D) show the P values from multiple regression of Z onto all traits in set A. The instrument in (A) and (B) was computed using an L1 penalty, while that in (C) and (D) was computed using an L2 penalty.

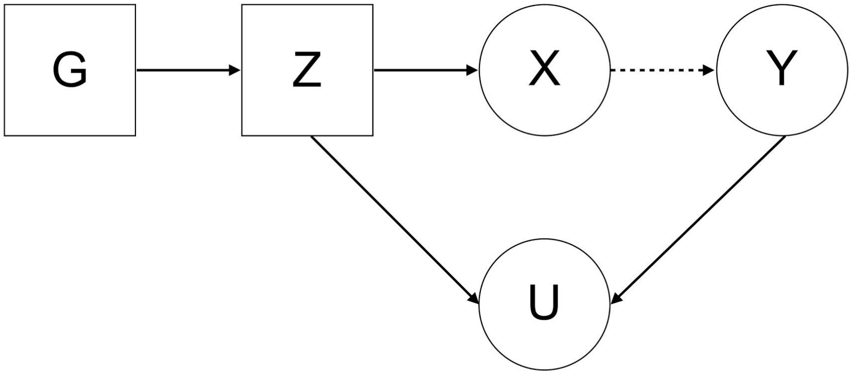

Figure S8. Example of collider bias. If Z and Y are independently causal for a variable U, then adjusting for U can induce an association between Z and Y that did not previously exist.

	All WES samples				Samples with WGS and WES data				WES samples excluding WGS samples			
	N	Beta	SE	P	N	Beta	SE	P	N	Beta	SE	P
**Fat mass**	11577	-0.094	0.010	5.79×10^{-23}	3016	-0.102	0.017	2.25×10^{-9}	8561	-0.091	0.011	3.01×10^{-15}
**ln(Fasting insulin)**	9434	-0.127	0.010	1.10×10^{-33}	2689	-0.135	0.019	1.98×10^{-12}	6745	-0.124	0.013	1.29×10^{-22}

Table S2. MT-CN associations with insulin and fat mass in WES data. Results of EMMAX test of association between normalized MT-CN and both fat mass and fasting serum insulin using WES data. Association tests were performed in all samples and also separately among samples with and without WGS data.

	Fat Mass			HDL			Total Triglycerides		
	Beta	SE	P	Beta	SE	P	Beta	SE	P
**Platelet count**	0.044	0.002	6.01×10^{-144}	-0.019	0.002	1.36×10^{-26}	0.088	0.002	$<2.22\times10^{-308}$
**Neutrophil count**	0.139	0.002	$<2.22\times10^{-308}$	-0.142	0.002	$<2.22\times10^{-308}$	0.179	0.002	$<2.22\times10^{-308}$
**Platelet count (no *post-hoc* outliers)**	0.044	0.002	5.58×10^{-144}	-0.019	0.002	1.19×10^{-26}	0.088	0.002	$<2.22\times10^{-308}$
**Neutrophil count (no *post-hoc* outliers)**	0.139	0.002	$<2.22\times10^{-308}$	-0.142	0.002	$<2.22\times10^{-308}$	0.179	0.002	$<2.22\times10^{-308}$

Table S3. Direct associations between platelet/neutrophil counts and metabolic syndrome phenotypes in the UK Biobank. Results of direct testing of platelet and neutrophil counts against metabolic syndrome phenotypes chosen based on marginally significant associations for MT-CN without adjustment for cell counts. Testing was done in the UK Biobank (N = 357,656) using linear regression of traits onto cell counts conditional on the same covariates as the MT-CN analyses (see Methods). The bottom two rows show the results after removal of high-residual, high-leverage outliers as determined by Cook's distance.

Cell type	rs2288464			rs9389268			*TMBIM1*
	Beta	SE	P	Beta	SE	P	P
**Leukocyte**	0.00921	0.00406	0.023	-0.0471	0.0027	4.85×10^{-66}	0.784
**Monocyte**	0.00670	0.00406	0.099	-0.0331	0.0027	2.18×10^{-33}	0.331
**Lymphocyte**	0.00525	0.00407	0.197	-0.0372	0.0028	1.65×10^{-41}	1
**Neutrophil**	0.00703	0.00405	0.083	-0.0363	0.0027	5.66×10^{-40}	0.274
**Platelet**	0.00714	0.00406	0.079	0.1109	0.0027	$<2.22\times10^{-308}$	0.082

Table S4. Associations in the UK Biobank between cell counts and loci mapped to MT-CN in Finnish data. Association tests in the UK Biobank (N = 357,656) of cell counts against common-variant and gene-based rare-variant associations found in Finnish GWAS. Single marker association statistics are calculated by linear regression, while *TMBIM1* P values are calculated by SKAT-O.

Dataset	n	Penalty	Beta	Std. Error	P
**WGS**	3034	L1	-0.019	0.013	0.1471
		L2	-0.020	0.011	0.0822
**Imputed Array**	6774	L1	-0.019	0.008	0.0188
		L2	-0.023	0.008	0.0045
**Meta-analysis**	-	L1	-0.019	0.007	0.0057
		L2	-0.022	0.007	0.0009

Table S9. Mendelian randomization results after removal of high leverage *post hoc* outliers. Results of Mendelian randomization test for causality of MT-CN on fasting serum insulin, after removal of high leverage *post hoc* outliers (see "Inference of causality in the association between MT-CN and insulin"). These results are nearly identical to those computed before outlier removal (**Figure 4d**), indicating that the detected signals were not driven by outlier samples.

Trait	SNP	GRCh37 coordinates	Ref.	Nearest gene(s)	METSIM MAF	METSIM P value	Notes
MT-CN	rs445	7:92408370	Cai, *et al*.[1]	CDK6	0.0504	0.04842	Lead marker for MT-CN in Cai, *et. al*.[1] and neutrophil count in Chen *et. al*.[2]
	rs11006126	10:60142880		TFAM	0.100	0.3609	
	rs709591	17:38175561	Guyatt *et al*.[3]	CSF3, MED24, PSMD3	0.402	1.61E-04	Not genome-wide significant in literature; MED24 is associated with neutrophil count (Chen et al.[2])
	rs12873707	13:104810437		LOC105370340	0.061	0.5048	Not genome-wide significant in literature
Neutrophil count	rs25645	17:38173143	Chen *et al*.[2]	CSF3	0.322	0.01712	2.5 kb away from rs709591
	rs2814778	1:159174683		ACKR1, CADM3-AS1	0.00056	0.6244	
	rs9131	4:74963049		CXCL2	0.355	0.04622	
	rs56388170	7:28724374		CREB5	0.299	0.009071	
	rs445	7:92408370		CDK6	0.0504	0.04842	Lead marker for MT-CN in Cai, *et. al*.[1] and neutrophil count in Chen *et. al*.[2]
Platelet count	rs11759553	6:135422296	Chen *et al*.[2]	HBS1L	0.348	2.15E-10	324 kb away from rs9389268, lead marker for MT-CN in METSIM
	rs9861033	3:56861222		ARHGEF3	0.339	0.2397	
	rs113608931	9:4758972		AL353151.2	0.301	0.3665	
	rs11066309	12:112883476		PTPN11	0.379	0.1081	
	rs549888	6:33552202		GGNBP1	0.453	0.1007	

Table S11. Replication in METSIM of published MT-CN QTLs. Results of GWAS of METSIM using imputed genotypes (N = 9,791) for top hits in the literature for MT-CN, neutrophil count, and platelet count. MT-CN loci were taken from references Cai, *et al*.[1] and Guyatt *et al.*[3]. Neutrophil and platelet count loci were chosen as the top 5 loci in the NHGRI-EBI GWAS Catalog for their respective traits, with some curation to ensure the loci tested were mutually independent. All ten variants came from either trans-ethnic or European-specific GWAS reported in reference Chen *et al*.[2]

Additional File 1 References

1. Cai N, Li Y, Chang S, Liang J, Lin C, Zhang X, et al. Genetic Control over mtDNA and Its Relationship to Major Depressive Disorder. Curr Biol. 2015;25:3170–7.

2. Chen M-H, Raffield LM, Mousas A, Sakaue S, Huffman JE, Moscati A, et al. Trans-ethnic and Ancestry-Specific Blood-Cell Genetics in 746,667 Individuals from 5 Global Populations. Cell. 2020;182:1198-1213.e14.

3. Guyatt AL, Brennan RR, Burrows K, Guthrie PAI, Ascione R, Ring SM, et al. A genome-wide association study of mitochondrial DNA copy number in two population-based cohorts. Hum Genomics. 2019;13:6.
