# Supplementary material for: Mitochondrial genome copy number measured by DNA sequencing in human blood is strongly associated with metabolic traits via cell-type composition differences
Source: Hum Genomics. 2021 Jun 7;15:34. doi: 10.1186/s40246-021-00335-2 (PMC8185936; doi:10.1186/s40246-021-00335-2)
Supplement: Supplementary file 3 — Additional file 3:. PDF format. Supplementary Methods. Describes methods in further detail than the main text. [file 40246_2021_335_MOESM3_ESM.pdf]

Supplementary Methods

Genotype and phenotype data

Genomic DNA was fragmented on the Covaris LE220 instrument targeting 375 bp inserts. Automated Illumina libraries were constructed with the TruSeq PCR-free (Illumina) or KAPA Hyper PCR-free library prep kit (KAPA Biosystems/Roche) on the SciClone NGS platform (Perkin Elmer). The fragmented genomic DNA was size-selected on the SciClone instrument with AMPure XP beads to tighten the distribution of fragmented DNA to ensure the average insert of the libraries was 350-375 bp. We followed the manufacturer's protocol as provided by Perkin Elmer, with the following exception: post ligation, the libraries were purified twice with a 0.7x AMPure bead/sample ratio to eliminate any residual adaptors present. An aliquot of the final libraries was diluted 1:20 and quantitated on the Caliper GX instrument (Perkin Elmer). The concentration of each library was accurately determined through qPCR utilizing the KAPA library Quantification Kit according to the manufacturer's protocol (KAPA Biosystems/Roche) to produce cluster counts appropriate for the Illumina HiSeqX instrument. Libraries were pooled and run over a few lanes of the HiSeq X to ensure the libraries within the pool were equally balanced. The final pool of balanced libraries was loaded over the remaining number of HiSeq X lanes to achieve the desired coverage for this project. 2x150 paired end sequence data were demultiplexed using a single index, which was a restriction on the HiSeqX instrument at this time. A minimum of 19.5x coverage was achieved per sample.

The quality of the aligned sequence data was assessed using metrics generated by Picard[1] v2.4.1, Samtools[2] v1.3.1 and VerifyBamID[3] v1.1.3. Based on

the output files from Picard, the following alignment statistics were collected for review: PF_MISMATCH_RATE, PF_READS, PF_ALIGNED_BASES, PCT_ADAPTER, PCT_CHIMERAS, PCT_PF_READS_ALIGNED, PCT_READS_ALIGNED_IN_PAIRS, PF_HQ_ALIGNED_BASES, PF_HQ_ALIGNED_Q20_BASES, PF_HQ_ALIGNED_READS, MEAN_INSERT_SIZE, STANDARD_DEVIATION, MEDIAN_INSERT_SIZE, TOTAL_READS, PCT_10x, and PCT_20x. Alignment rate was calculated as PF_READS_ALIGNED/TOTAL_READS. The formula for haploid coverage was as follows: $Haploid\ coverage = MEAN\_COVERAGE * \frac{1-PCT\_EXC\_DUPE}{1-PCT\_EXC\_TOTAL}$. From the Samtools output, inter-chromosomal rate was calculated as: $\frac{reads\_mapped\_in\_interchromosomal\_pairs}{reads\_mapped\_in\_pair}$ and discordant rate was calculated as: $reads\_mapped\_percentage - reads\_mapped\_in\_proper\_pairs\_percentage$.

Properly paired percentage (reads_mapped_in_proper_pairs_percentage) and singleton percentage (reads_mapped_as_singleton_percentage) were also reviewed. From VerifyBamID, the Freemix value was reviewed.

The metrics for judgement of passing data quality were: FIRST_OF_PAIR_MISMATCH_RATE < .05, SECOND_OF_PAIR_MISMATCH_RATE < 0.05, haploid coverage ≥ 19.5, interchromosomal rate < .05, and discordant rate < 5. All of the above metrics must have been met in order for the sample to be assigned as QC pass. If a sample did not meet the passing criteria, the following failure analysis was performed: a) If the Freemix score was at least 0.05, the sample or the library was considered contaminated, and both the library and the sample were abandoned; b) if the discordant rate was over 5 and/or the inter-chromosomal rate was over 0.05, the quality of DNA was considered poor and the sample was removed from the sequencing pipeline;

and c) in the case of a) and b), the collaborator was contacted to determine if selection of a replacement sample from the same cohort was desired or feasible.

WGS callset generation and quality control

Single nucleotide polymorphisms (SNPs) and small insertions and deletions were called from the full set of 4,163 samples using GATK[4] v3.5. GVCFs containing SNVs and Indels from GATK HaplotypeCaller (`-ERC GVCF -GQB 5 -GQB 20 -GQB 60 -variant_index_type LINEAR -variant_index_parameter 128000`) were first processed to ensure no GVCF blocks crossed boundaries every 1 Mb (CombineGVCFs; `--breakBandsAtMultiplesOf 1000000`). The resulting GVCFs were then processed in 10 Mb shards across each chromosome. Each shard was combined (CombineGVCFs), genotyped (GenotypeGVCFs; `-stand_call_conf 30 -stand_emit_conf 0`), hard filtered to remove alternate alleles uncalled in any individual removed (SelectVariants; `--removeUnusedAlternates`), and hard filtered to remove lines solely reporting symbolic deletions in parallel. All shards were jointly recalibrated (VariantRecalibrator) and then individually filtered (ApplyRecalibration) based on the recalibration results. All of the above methods were performed using GATK v3.5. SNP variant recalibration was performed using the following options to VariantRecalibrator and all resources were drawn from the GATK hg38 resource bundle (v0):

```
-mode SNP
-resource:hapmap,known=false,training=true,truth=true,prior=15.0
-resource:omni,known=false,training=true,truth=true,prior=12.0
-resource:1000G,known=false,training=true,truth=false,prior=10.0
-resource:dbsnp,known=true,training=false,truth=false,prior=2.0
-an QD -an DP -an FS -an MQRankSum -an ReadPosRankSum
```

```
-tranche 100.0 -tranche 99.9 -tranche 99.0 -tranche 90.0
```

Indel variant recalibration was performed using the following options to VariantRecalibrator (with the same resource bundle as with SNPs):

```
-mode INDEL
-resource:mills,known=true,training=true,truth=true,prior=12.0
-an DP -an FS -an MQRankSum -an ReadPosRankSum
--maxGaussians 4
-tranche 100.0 -tranche 99.9 -tranche 99.0 -tranche 90.0
```

When applying the variant recalibration the following options were used:

For SNPs: `--ts_filter_level 99.0`

For Indels: `--ts_filter_level 99.0`

Following SNP and INDEL variant recalibration, multiallelic variants were decomposed and normalized with vt[5] v0.5. Duplicate variants and variants with symbolic alleles were subsequently removed. The bottom tranche of variants identified by GATK's Variant Quality Score Recalibration tool and variants with missingness greater than 2% were removed as well, although variants with allele balance between 0.3 and 0.7 were rescued. Variants with Hardy-Weinberg equilibrium (on a second-degree unrelated subset of 3,969 individuals, as determined by KING[6]) P value less than 10^{-6} and those with allele balance less than 0.3 or greater than 0.7 were also removed.

Sample-level quality control was also undertaken on this dataset; 13 samples were identified for exclusion because of singleton counts that were at least eight median absolute deviations above the median. Separately, 12 sex-discordant samples were flagged using `plink --check-sex`, and after examining chromosome Y missingness

and F coefficient values for these samples, only the one that clearly differed from its reported sex was marked for exclusion. No samples were excluded based on missingness fraction or the first five principal components. In total, 14 samples were excluded from the heritability, GWAS, and Mendelian randomization analyses; the other analyses were performed without exclusion of these samples. As a result, the former analyses were performed with N = 4,149 while the latter had N = 4,163.

Mendelian Randomization

In our formulation (**Figure 4a**), X, the natural log of MT-CN (adjusted for nuclear genomic coverage but not for age, age^2, or sex), and a genotype matrix G were used to build a genetic instrument Z, which was then tested against Y, the natural log of fasting serum insulin. The goal of the MR approach was to use a large number of common variants to build a genetic instrument Z that satisfies the three assumptions of MR[7]:

1. Association of Z with X
2. Independence of Z from any variables U confounding the relationship between X and Y
3. Independence of Z and Y given X and U

To attempt to build a genetic instrument satisfying assumptions 2 and 3 (see Methods), the deep METSIM phenotype data were leveraged. A matrix W was constructed using the 75 measured traits and first 20 PCs of the genotype matrix (including a third-degree polynomial basis for PC 1). From these variables, covariates that could violate one of these two assumptions were chosen by selecting columns of W associated with X or Y (**Figure 4b**). These columns were selected using two successive LASSO feature selection procedures. First, a set A of covariates associated with Y was

chosen by using LASSO to regress Y onto W. In this regression, age and the third-degree polynomial basis for PC 1 were left unpenalized to ensure that A contains these covariates. The shrinkage parameter was chosen by tenfold cross-validation as the largest value that gives a mean squared error (MSE) within one standard error of the minimum observed MSE. Next, the columns of W associated with X conditional on A were chosen using a similar LASSO procedure in the regression of X onto W. In this step, however, the variables in set A were left unpenalized in order to only capture associations that are conditionally independent of A. The selected variables from this regression were designated set B.

The instrument was built using a penalized regression (using either an L1 or L2 penalty, as implemented in glmnet[8]) of the form $X \sim G + W_A + W_B$, where $\mathrm{W_A}$ and $\mathrm{W_B}$ are the columns of W representing sets A and B, respectively, and G is a genotype matrix containing the alternate allele dosage (missing alleles are replaced with the MAF, similarly to PLINK[9]) of all variants with MAF greater than 1% and marginal GWAS P value below 0.01. As X was the target vector for this regression, assumption 1 of MR was trivial. In the penalized regression, $\mathrm{W_A}$ and $\mathrm{W_B}$ were unpenalized in an effort to orthogonalize the regression coefficients of the genotypes to these covariates in an effort to enforce assumptions 2 and 3. glmnet was run with a convergence threshold of 1×10^{-10} and maximum number of iterations of 200,000. To avoid the overfitting that would result from calculating instrument values on the same samples on which regression coefficients are learned[10], the penalized regression model was fit on independent subsets of the data as follows. Five models were fit, each by holding out a different 20% of samples, such that the instrument value computed for each sample was calculated using the regression

coefficient vector learned without that sample. The vector of possible shrinkage parameters λ for all five models was supplied as (10^3,10^2,…,10^{-13},10^{-14}), and the λ value which minimized the joint residual sum of squares of all five models was chosen for instrument calculation.

Formally, we randomly partitioned the set of samples S with nonmissing insulin measurements into five nonoverlapping sets S_j for $j = \{1, \ldots, 5\}$. We denote set complements as $S_j^C = S \setminus S_j$, such that each S_j^C contained 80% of the training samples. The instrument vector Z_j for each S_j was computed as follows: $Z_j = G_j \times \beta_G^{(-j)}$, where Z_j is the instrument vector for S_j, G_j is the genotype matrix of S_j, and $\beta_G^{(-j)}$ is the vector of genotype regression coefficients from the model described above, trained on S_j^C. The instrument values within each S_j were inverse rank-normalized using a Blom transformation[11,12] before being concatenated across the values of j to give the final instrument vector Z. Because samples with missing insulin values could not be included in the causality test anyway, these samples were excluded from S but safely included in the training sets of all five models. The instrument values of these samples were never calculated or used in downstream analyses.

Often, the inclusion of unpenalized covariate sets A and B in the instrument-building regression was not sufficient to completely orthogonalize Z to these covariates (see below). As a result, the test for association between Z and Y was performed conditional on a set of potentially assumption-violating covariates chosen using the newly constructed instrument Z in another attempt to account for possible violations of MR assumptions in the causality test (**Figure 4c**). To choose this set of covariates C, a final feature selection step was performed using LASSO regression of Z on W with covariate

set A excluded from the penalty. As in the previous feature selection steps, the shrinkage parameter was chosen via tenfold cross-validation as the largest value with MSE within one standard error of the minimum observed MSE. Once this set, D, of covariates associated with Z was chosen, the covariates in W were partitioned into sets I, II, III, and IV based on their membership in A and D (see **Figure 4**). Formally, this partitioning was done as follows: $I = W \setminus (A \cup D)$, $II = D \cap A^C$, $III = A \cap D^C$, and $IV = A \cap D$, where $A^C = W \setminus A$ and $D^C = W \setminus D$. Then, the test for causality came from the regression coefficient of Z in the multiple regression $Y \sim Z + C$, where C is the union of sets II, III, and IV (colored blue in **Figure 4c**).

Additional File 3 References

1. Picard Toolkit [Internet]. Broad Institute, GitHub repository; 2019. Available from: http://broadinstitute.github.io/picard/

2. Li H, Handsaker B, Wysoker A, Fennell T, Ruan J, Homer N, et al. The Sequence Alignment/Map format and SAMtools. Bioinformatics. 2009;25:2078–9.

3. Jun G, Flickinger M, Hetrick KN, Romm JM, Doheny KF, Abecasis GR, et al. Detecting and estimating contamination of human DNA samples in sequencing and array-based genotype data. Am J Hum Genet. 2012;91:839–48.

4. DePristo MA, Banks E, Poplin R, Garimella KV, Maguire JR, Hartl C, et al. A framework for variation discovery and genotyping using next-generation DNA sequencing data. Nat Genet. 2011;43:491–8.

5. Tan A, Abecasis GR, Kang HM. Unified representation of genetic variants. Bioinformatics. 2015;31:2202–4.

6. Manichaikul A, Mychaleckyj JC, Rich SS, Daly K, Sale M, Chen W-M. Robust relationship inference in genome-wide association studies. Bioinformatics. 2010;26:2867–73.

7. Palmer TM, Lawlor DA, Harbord RM, Sheehan NA, Tobias JH, Timpson NJ, et al. Using multiple genetic variants as instrumental variables for modifiable risk factors. Stat Methods Med Res. 2012;21:223–42.

8. Friedman J, Hastie T, Tibshirani R. Regularization Paths for Generalized Linear Models via Coordinate Descent. J Stat Softw. 2010;33:1–22.

9. Chang CC, Chow CC, Tellier LC, Vattikuti S, Purcell SM, Lee JJ. Second-generation PLINK: rising to the challenge of larger and richer datasets. Gigascience. 2015;4:7.

10. Burgess S, Thompson SG. Use of allele scores as instrumental variables for Mendelian randomization. Int J Epidemiol. 2013;42:1134–44.

11. Beasley TM, Erickson S, Allison DB. Rank-based inverse normal transformations are increasingly used, but are they merited? Behav Genet. 2009;39:580–95.

12. Blom G. Statistical estimates and transformed beta-variables [Internet]. Almqvist & Wiksell; 1958 [cited 2020 Jun 5]. Available from: http://www.diva-portal.org/smash/record.jsf?pid=diva2:516729
